# Advances in Retinal Therapeutics

**DOI:** 10.1155/2015/515193

**Published:** 2015-03-09

**Authors:** Petros E. Carvounis, Thomas A. Albini, Andrew J. Barkmeier, Miltiadis Tsilimbaris

**Affiliations:** ^1^Cullen Eye Institute, Baylor College of Medicine, Houston, TX 77030, USA; ^2^Bascom Palmer Eye Institute, Miami, FL 33136, USA; ^3^Department of Ophthalmology, Mayo Clinic, Rochester, MN 55905, USA; ^4^Department of Ophthalmology, University of Crete Medical School, 71 003 Heraklion, Greece

In recent years there have been monumental and exciting advances in the treatment of retinal disease which made a special issue on advances in retinal therapeutics relevant and interesting. Indeed, in this issue there are 11 papers reporting either original basic or clinical research findings or reviewing the literature and reporting recent developments.

The papers in this issue are on a wide range of topics. J. He et al. present original basic research in elucidating the effects of VEGF receptor 1 blockade on diabetic retinopathy while M. L. Perepechaeva et al. present original basic research identifying a potential novel target for the treatment of age-related macular degeneration in a rodent animal model of the disease. O. Chrapek et al. present original clinical research identifying occult CNVM and lesion size less than 5 disc diameters as baseline characteristics predicting inactivity after 3 injections of an intravitreous anti-VEGF agent (ranibizumab) for neovascular age-related macular degeneration (nARMD). S. M. Prea et al. review endogenous inhibitors of nARMD as well as the principles of gene therapy that could use such inhibitors to treat nARMD clinically. There are 2 papers discussing advances in retinal laser treatment—one by Y. G. Park et al. discussing developments on laser photocoagulation for diabetic macular edema and another by K. Inagaki et al. discussing micropulse laser for persistent macular edema from branch retinal vein occlusion in eyes which includes eyes with good visual acuity at baseline. Two additional papers address vitreoretinal surgery topics: one by Dr. C. Pournaras et al. that reports on the outcomes of repair of recurrent retinal detachment, including presenting visual outcomes which are not uncommonly omitted in prior publications and the other paper by A. García-Layana et al. that reviews the current treatment of vitreomacular traction and macular hole. The paper by M. Harrell and P. E. Carvounis is an up-to-date evidence-based review of the treatments of toxoplasma retinochoroiditis which takes into account evidence published within the last 2 years when the previous evidence-based review was published. Finally, the paper by M. Cabrera et al. reviews the 3 sustained-release corticosteroids available for the treatment of retinal disease.

The 54 authors and coauthors should be commended for the quality of their manuscripts; the science as well as the writing is of high caliber. It is our belief that the readership of the Journal of Ophthalmology will enjoy reading the papers in this special issue as much as we did.

*Petros E. Carvounis* *Petros E. Carvounis* *Thomas A. Albini* *Thomas A. Albini* *Andrew J. Barkmeier* *Andrew J. Barkmeier* *Miltiadis Tsilimbaris* *Miltiadis Tsilimbaris*

